# Socioeconomic Inequalities in Visits to the Dentist to Receive Professionally Applied Topical Fluoride in a Developing Country

**DOI:** 10.3390/ijerph14080903

**Published:** 2017-08-11

**Authors:** Miriam Del Socorro Herrera, Carlo Eduardo Medina-Solís, América Patricia Pontigo-Loyola, Rubén de la Rosa-Santillana, Leticia Ávila-Burgos, Rogelio José Scougall-Vilchis, Sonia Márquez-Rodríguez, Mirna Minaya-Sánchez, Alejandro José Casanova-Rosado

**Affiliations:** 1 Faculty of Dentistry of National Autonomous, University of Nicaragua, León 21000, Nicaragua; miriam.sherrera@yahoo.com; 2 Academic Area of Dentistry of Health Sciences Institute at Autonomous, University of Hidalgo State, Pachuca 42039, Mexico; americap@uaeh.edu.mx (A.P.P.-L.); cdrubendelarosa@hotmail.com (R.d.l.R.-S.); cdsoniamr@hotmail.com (S.M.-R.); 3 Advanced Studies and Research Center in Dentistry “Keisaburo Miyata” of Faculty of Dentistry at Autonomous, University State of Mexico, Toluca 50000, Mexico; rogelio_scougall@hotmail.com; 4 Health Systems Research Centre at National Institute of Public Health, Cuernavaca 62100, Mexico; leticia.avila@insp.mx; 5 Faculty of Dentistry of Autonomous, University of Campeche, Campeche 24039, Mexico; miminaya@hotmail.com (M.M.-S.); ajcasano@uacam.mx (A.J.C.-R.)

**Keywords:** oral health, health services, caries prevention, topical fluoride, Nicaragua

## Abstract

Objective: To determine the frequency and associated factors of visits to the dentist in the last year by Nicaraguan schoolchildren to receive professionally applied topical fluoride (PATF). Material and Methods: A cross-sectional study was designed in children from public schools in the city of León, Nicaragua, were included. A series of socioeconomic, sociodemographic, and behavioural variables were collected through a questionnaire. The dependent variable was the visit to the dentist to receive professionally applied topical fluoride in the last year, which was dichotomised as (0) Did not receive PATF and (1) Yes received PATF. In the statistical analysis, binary logistic regression was used. Results: The mean age of the students included was 9 years, and 49.9% were girls. The prevalence of visits to the dentist in the last year to receive PATF was 3.1%. In the multivariate model, the associated characteristics (*p* < 0.05) were: female (OR = 2.73, 95% CI = 1.34–4.50); the positive attitude of the mother to the oral health of her child (OR = 2.15, 95% CI = 1.03–4.50); and the best socioeconomic position (OR = 2.68, 95% CI = 1.36—5.31). Conclusions: The prevalence of visits to the dentist in the last year to receive professionally applied topical fluoride was very low (3.1%). The results of the socioeconomic position suggest the existence of certain inequalities in oral health. It is necessary to implement policies and programs aimed at improving this scenario.

## 1. Introduction

Socioeconomic inequalities in health refer to the different health-related opportunities and resources that people of different social classes have, so that the most disadvantaged groups are in worse health than their better-positioned counterparts. These differences have their origin in the inequalities of the economic and social policies that exist in society [1]. In this sense, studies carried out in several countries around the world, both developed and developing, show the existence of socioeconomic inequalities in various aspects of health. It is observed that the socioeconomically disadvantaged population is generally the one with the lowest levels of health [2,3,4].

Dental caries continues to be one of the main oral health problems worldwide in school-aged children, and are concentrated in the most disadvantaged social groups [5]. Pitts et al. [6] mention that there are marked differences in caries-related health inequalities in developing and developed countries. Also, it is evident that there is a gradient in caries levels between the least developed and the most developed countries. According to epidemiological studies, in Nicaragua, the prevalence of dental caries in primary dentition is 77.7%, with an average dmft (decayed, missing and filled teeth) index of 3.54 ± 3.13 for children 6 to 9 years of age [7]. In permanent dentition the prevalence is 37.9%, with a mean DMFT (Decayed, Missing and Filled teeth) index of 0.98 ± 1.74 [8]. At the same time, it is observed that little more than 50% of caries lesions in the permanent dentition present treatment needs [9]. Likewise, there are differences between the different social groups, suggesting certain socioeconomic inequalities in oral health in the country. This disease represents a substantial burden both for health systems and for households, due to the costs required for their treatment; the more severe the lesion, the greater the technology and the resources required, so families often incur direct out-of-pocket expenses to obtain the needed services to maintain adequate oral health [10]. According to the World Health Organization, there is limited availability or inaccessibility of oral health services, which makes their utilisation rates particularly low among some vulnerable groups of the population. Moreover, it is estimated that in high-income countries, traditional curative dental care consumes between 5% and 10% of public health expenditure [11].

At the community level, as well as in clinical practice, one of the widely-used technologies to prevent and treat dental caries in its early stages is fluoride [12]; in addition, there are several modes available to provide it professionally, for example, varnish, gel, and fluoride solution [13,14,15]. In developed countries, the use of fluoride and gel varnish is common among dentistry professionals. The relatively high cost of professionally administered fluoridated agents and the shortage of dental labour, especially in low-income countries, have hindered the widespread adoption of these methods in public and private dental services [16]. In addition to this scenario, one of the most important barriers preventing people from going to health services is their economic type [17].

Recently, several studies have analysed the impact of the socioeconomic position on the oral health of the population, which is strongly determined by this variable, and is referred to as inequality in oral health. The exact path by which oral health or utilisation of oral health services and socioeconomic position are associated is still unclear. Likely mechanisms involve healthy behaviours, healthy lifestyles, and health care. In fact, the association between socioeconomic status and health is so strong that several researchers have hypothesised that social position may be the root cause of health [18]. Also, inadequate access to essential health services is one of the several determinants of social inequities in health. It may not be the major determinant, but it is important for the health sector to tackle directly [19]. The factors associated with the demand for professionally-applied topical fluoride (PATF) have been studied little, and in Nicaragua, there is no scientific antecedent of the subject. Studies in other countries have observed that the variables, associated with requests for dentist-administered PATF are: the best socioeconomic position [20,21], the greater frequency of dental brushing [20], the age of the children, greater parental knowledge about oral health, greater brushing frequency of parents, and access to some type of health insurance [21], among others. So, the objective of the present study is to determine the frequency of visits of Nicaraguan children to the dentist to receive PATF in the past year and the factors associated with these visits.

## 2. Materials and Methods

### 2.1. The Nicaraguan Health System Context

Nicaragua, after Haiti, is the second poorest nation in the region. The General Health Law in Nicaragua establishes three pillars of the National Single Health System: the contributory, the non-contributory, and the voluntary. The System has public and private participation in the financing and provision of services, with little integration and articulation of the subsectors. The Ministry of Health (MINSA) is responsible for the Sectoral Rectory and is the primary service provider. The contributory scheme includes the Nicaraguan Social Security Institute (INSS), which administers, among other things, compulsory and optional health insurance for workers in the formal sector and their families, and the Ministries of Governance (MIGOB) and Defence (MIDEF) which cover their employees and their families. The non-contributory regime is in charge of the Ministry of Health and covers population groups without payment capacity. The voluntary scheme serves groups with payment capacity that purchase services with direct payment and the non-paying population within the non-profit private sector, which includes civil society and non-governmental organisations. Within the public sector, the Ministry of Health is the main provider. In 2008, the INSS covered 16.5% of the population; the MIGOB and the MIDEF 6%; and the MINSA 61.2%. The MINSA, the MIDEF, and the MIGOB have their own clinics and hospitals [22].

### 2.2. Study Design and Sample Selection

We used data from a cross-sectional study of which other aspects related to oral health were previously published [7,8,9,23]. The study universe consisted of 18,574 schoolchildren aged 6 to 12 years from elementary schools in the city of León, Nicaragua. Inclusion criteria were: (1) children apparently healthy aged 6 to 12 years; (2) enrolled in selected elementary schools; (3) whose parents gave informed consent for their children to participate in the study; and (4) who did not have fixed orthodontic appliances. Of the total number of existing schools in Leon, Nicaragua, through a simple random sampling, 25 schools were selected. Subsequently, using the sampling strategy proposed by the WHO [24], four boys and four girls from each of the seven age groups were included, which, in the end, resulted in 56 children from each of the 25 schools. The consent rate was >90%. In this way, they were similarly distributed by age and sex, which gave a final sample of 1400 schoolchildren.

### 2.3. Variables and Data Collection

The dependent variable was a visit to the dentist to receive PATF in the last year, which was dichotomised as (0) those who did not visit the dentist to receive PATF; and (1) those who did visit the dentist to receive PATF. The independent variables were collected through a questionnaire containing questions describing socioeconomic, sociodemographic, and behavioural variables. The variables included in the study were age, sex, birth order, and frequency of brushing; age, schooling, and occupation of the parents were also included, in addition to family size and the attitude of the mother toward oral health. The importance attributed by the mother to the oral health of the children was reduced to a positive attitude (1) if she answered “yes” to the following two questions: *Do you consider it important that your child’s teeth are kept in good condition? Have you ever examined your child's teeth to determine if they were healthy?* A negative attitude (0) was registered if there was a “no” response to either of these two questions. This approach has been used in several studies [25,26].

Indicators of socioeconomic position in the study. With the schooling and the occupation of both parents, two variables were established indicating socioeconomic position. For its construction, the analysis of principal components was used; specifically, that of polychoric correlation [27]. The two variables obtained in this way and related to each of these dimensions (schooling and occupation) were categorised into tertiles, where the first tertile refers to the worst socioeconomic position and the third tertile to the best.

### 2.4. Analysis of Data

A univariate analysis was performed in which measures of central tendency and dispersion were calculated for the continuous variables. In the case of categorical variables, frequencies were obtained for each category, as well as the corresponding percentage. For the bivariate analysis, chi-square and Mann-Whitney tests were used according to the measurement scale of the variables to be tested.

In the multivariate model, binary logistic regression was used. For the construction of the final model, the steps marked by the literature were followed [28]. The final model included variables that had a value of *p* < 0.25 in the bivariate analysis. An analysis of the variance inflation factor (VIF) was performed to detect and, if necessary, avoid multicollinearity between the independent variables. In both bivariate and multivariate analyses, the confidence intervals were calculated with robust standard errors (Huber-White errors), which accommodates accurate estimates even in cases of correlation by groups (schools). This is because the data observed were from children from different schools. Although this allows correlation within the same, it also suggests statistical independence between them. The program used for statistical procedures was STATA 11.0.

### 2.5. Ethical Considerations

This study was carried out according to the general health law in research and the scientific principles of the Declaration of Helsinki. Written consent was obtained from the parents/guardians of the participating children. The protocol was approved by the ethics committee review board of the Universidad Nacional Autónoma de Nicaragua (Campus León) and the Universidad Autónoma de Campeche (UAC/FO/MC-005), where one of the authors earned his Master of Science degree.

## 3. Results

In the study were included 702 boys and 698 girls with a mean age of 8.99 ± 2.00 years. The descriptive results are shown in Table 1. Most mothers demonstrated a positive attitude towards their children’s oral health (57.1%); the mean age of the mothers was 33.07 ± 6.05 years. The prevalence of visits to the dentist in the last year to receive PATF was 3.1%.

Table 2 shows the results of the bivariate analysis. Statistically significant differences (*p* < 0.05) were observed through the categories of variables: sex, mother’s attitude towards the child’s oral health, dental brushing frequency, and socioeconomic position (scholarship).

In the multivariate model of binary logistic regression (Table 3), it was observed that women were more likely than men to have visits to the dentist in the last year to receive PATF (OR = 2.73, 95% CI = 1.34–4.50). The children of those mothers who had a positive attitude toward their child’s oral health presented 2.5 times greater (95% CI = 1.03–4.50) odds of visits to the dentist in the last year to receive PATF than those of mothers with negative attitudes. Finally, the best socioeconomic position (OR = 2.68, 95% CI = 1.36–5.31) increased the odds of having visits to the dentist in the last year to receive PATF.

## 4. Discussion

In this study, we identified several variables associated with visits to the dentist in the last year to receive professionally applied topical fluoride; in addition, its prevalence was only 3.1%, which could be considered low when it is known to be one of the most effective measures to reduce the prevalence of caries, especially in a developing country. In Nicaragua, as in other Latin American countries [29,30,31,32,33], oral health among school-age children shows unfavourable outcomes. There is a high prevalence of dental caries, high unmet oral health needs, and low experience of restorative treatment. Added to this scenario, as in other parts of the world [34], the use of preventive oral health services, in the application of PATF, is very low. In the world literature, very few studies have been done on the subject, since the majority are studies on the effectiveness of dental caries prevention. According to these studies, the work carried out in other countries indicates that the prevalence is between 5.1% and 11.5% [20,21], higher than that reported in the present study. Although there is no currently pure scientific consensus on the periodicity of visits to the dentist, the American Academy of Pediatrics and the American Academy of Pediatric Dentistry recommend the age of onset for the first consultation for a review of routine and to check the state of oral health of children by their first birthday, with subsequent periodic reviews at least twice a year. With these actions, the opportunity to perform both preventive and curative treatments of minimal invasion among children is increased [35,36]. On the other hand, a series of meta-analyses carried out by Marinho et al. on the application of fluoride in rinses [13], gel [14], and varnishes [15], conclude that there is clear evidence that the regular use of fluoride in one of these presentations has an effect on the reduction of caries in the primary and permanent dentition. Additionally, according to these recommendations, several studies have shown that regular care is associated with better oral health responses and a better quality of life [37,38].

Compared with men, women realise the greater use of health services. However, the increased use of health services by women is not a consistent finding, but depends, in part, on the type of service. Thus, women tend to use preventive and diagnostic services more frequently, while men make greater use of emergency services [39,40,41]. A similar pattern is observed in relation to oral health services [42]. In this sense, the results of the present study show that women also performed a higher percentage of visits to the dentist to receive PATF.

Other studies [26,43,44] have observed that maternal characteristics are related to the use of oral health services. Specifically, the attitude of the mother to the oral health of her child has been pointed out by Andersen & Davidson [45], who document the importance of attitudes toward health as strong predictors of the use of health services.

In the present study, we included several variables that theoretically determine the socioeconomic position. According to Galobardes et al. [46], schooling is an indicator of socioeconomic position that attempts to capture the stock of knowledge of a person. As formal education is usually completed in early adulthood and is strongly determined by the socioeconomic characteristics of parents, it can be conceptualised within the framework of the “life course.” On the other hand, occupation is a measure of socioeconomic position that is frequently used, and represents the place of a person within society by income and intellect, as well as characterising the working relationships between employers and employees. The occupation of the “head of the household”, or the “highest occupation within the household”, can be used as a socioeconomic position indicator of dependents (e.g., spouse, children) or the house as a unit. Family size is a variable that has an influence on the socioeconomic position. Based on what Mechanic calls “competitive needs at home”, the greater the number of individuals in the household, the greater the competition between them for family resources [47]. In our study, the variable that was related to visits to the dentist to receive PATF was schooling.

Despite the large number of studies that have addressed a range of social groups and health services (including preventive services), there is still little understanding of the underlying mechanisms that drive the persistence of socio-economic inequalities in the use of preventive services. Recently, the cultural capital of health and healthy lifestyles have been discussed theoretically concerning their role in health inequalities in the case of preventive care [48]. Specifically, inequalities in the use of preventive dental services have been observed in Spain [49] and, according to the present study, our results suggest the existence of inequalities in oral health.

The present analysis has limitations that must be considered for a better interpretation of the results. For example, the data are derived from a cross-sectional study, where the problem of temporal ambiguity is common, which refers to the measurement of cause and effect at the same time so those causal relationships cannot be established. Another limitation is that in retrospective studies, there is the possibility of introducing recall bias. 

## 5. Conclusions

Based on the observed results, we can conclude that the prevalence of visits to the dentist in the last year to receive PATF was very low (only 3.1%) when compared to the results obtained in other studies. It is known that PATF is one of the most effective measures to reduce the prevalence of caries, especially in a developing country. The results of the socioeconomic position suggest the existence of certain inequalities in oral health to this indicator. It is necessary to implement policies and programs aimed at improving this scenario.

## Figures and Tables

**Table 1:** Descriptive results of study sample.

Variable	Frequency	Mean ± SD; (Limits)
Age	1400	8.99 ± 2.00; (6–12)
Mother’s age	1400	33.07 ± 6.05; (20–52)
Family size (number of children)	1400	3.12 ± 1.58; (1–12)
		Percentage
Sex		
Men	702	50.1
Women	698	48.8
Birth order		
First to third	1166	83.3
Fourth or more	234	16.7
Mother’s attitude towards oral health		
Negative	600	42.9
Positive	800	57.1
Frequency of tooth brushing		
Less than 7 times/week	676	48.3
At least 1 time per day	724	51.7
SEP (occupation)		
Low	561	40.1
Medium	526	37.6
High	313	22.4
SEP (schooling)		
Low	507	36.2
Medium	436	31.1
High	457	32.6

**Table 2:** Bivariate results for visits to the dentist for PATF in children from 6 to 12 years of age.

Variable	Without PATF	With PATF	*p* Value
Age	8.99 ± 2.00	9.36 ± 1.96	0.2199 *
Mother’s age	33.07 ± 6.10	32.98 ± 5.11	0.9083 *
Family size (number of children)	3.13 ± 1.59	2.72 ± 1.18	0.1080 *
Sex			
Men	689 (98.1)	13 (1.9)	
Women	667 (95.6)	31 (4.4)	0.005 †
Birth order			
First to third	1127 (96.7)	39 (3.3)	
Fourth or more	229 (97.9)	5 (2.1)	0.334 †
Mother’s attitude towards oral health			
Negative	589 (98.2)	11 (1.8)	
Positive	767 (95.9)	33 (4.1)	0.015 †
Frequency of tooth brushing			
Less than 7 times/week	665 (98.4)	11 (1.6)	
At least 1 time per day	691 (95.4)	33 (4.6)	0.002 †
SEP (occupation)			
Low	547 (97.5)	14 (2.5)	
Medium	505 (96.0)	21 (4.0)	
High	304 (97.1)	9 (2.9)	0.351 †
SEP (schooling)			
Low	498 (98.2)	9 (1.8)	
Medium	426 (97.7)	10 (2.3)	
High	432 (94.5)	25 (5.5)	0.002 †

* Mann-Whitney; † Chi-square.

**Table 3:** Multivariate logistic regression model for visits to the dentist in the last year by Nicaraguan schoolchildren to receive professionally applied topical fluoride.

Variable	OR	95% CI	*p* Value
Sex			
Men	1 *		
Women	2.73	1.34–5.56	0.006
Mother’s attitude towards oral health			
Negative	1 *		
Positive	2.15	1.03–4.50	0.042
SEP (schooling)			
Low/Medium	1 *		
High	2.68	1.36–5.31	0.005

* Reference category. Note: Model adjusted for the variables in the table as well as by age. 95% CI estimated with robust standard errors (cluster school). Goodness-of-fit test: Hosmer y Lemeshow: chi2 = 10.64, *p* = 0.2228.
